# Accelerating Image Reconstruction in Dual-Head PET System by GPU and Symmetry Properties

**DOI:** 10.1371/journal.pone.0050540

**Published:** 2012-12-26

**Authors:** Cheng-Ying Chou, Yun Dong, Yukai Hung, Yu-Jiun Kao, Weichung Wang, Chien-Min Kao, Chin-Tu Chen

**Affiliations:** 1 Department of Bio-Industrial Mechatronics Engineering, National Taiwan University, Taipei, Taiwan; 2 Department of Biomedical Engineering, Illinois Institute of Technology, Chicago, Illinois, United States of America; 3 Department of Mathematics, National Taiwan University, Taipei, Taiwan; 4 Department of Radiology, The University of Chicago, Chicago, Illinois, United States of America; Banner Alzheimer's Institute, United States of America

## Abstract

Positron emission tomography (PET) is an important imaging modality in both clinical usage and research studies. We have developed a compact high-sensitivity PET system that consisted of two large-area panel PET detector heads, which produce more than 224 million lines of response and thus request dramatic computational demands. In this work, we employed a state-of-the-art graphics processing unit (GPU), NVIDIA Tesla C2070, to yield an efficient reconstruction process. Our approaches ingeniously integrate the distinguished features of the symmetry properties of the imaging system and GPU architectures, including block/warp/thread assignments and effective memory usage, to accelerate the computations for ordered subset expectation maximization (OSEM) image reconstruction. The OSEM reconstruction algorithms were implemented employing both CPU-based and GPU-based codes, and their computational performance was quantitatively analyzed and compared. The results showed that the GPU-accelerated scheme can drastically reduce the reconstruction time and thus can largely expand the applicability of the dual-head PET system.

## Introduction

Positron emission tomography (PET) is a proven molecular-imaging technology for a wide range of biomedical researches and applications [1]–[5]. As its use widens and increases, it has been recognized that both the resolution and sensitivity of PET imaging need to be considerably improved, especially for small-animal imaging applications [6]–[8]. Dedicated small-animal PET systems that employ High Resolution Research Tomograph (HRRT) detectors can reach good spatial resolution and improved sensitivity. In addition to providing high detection sensitivity and cost effectiveness, planar PET imaging is particularly suitable for imaging thin and small objects like plant leaves. The growing interest in these extended applications also inspired development of PET detectors designed specifically for plants [9], [10]. However, this is a challenging goal due to the so-called depth-of-interaction (DOI) blurring that leads to reduced image resolution when thick scintillators or compact scanner geometry are used for increasing sensitivity. To address the issue of depth-of-interaction (DOI) blurring, thick detectors [11]–[18] and accurate image reconstruction methods based on physical and statistical models [19]–[23] have been developed. Despite these extended applications and improvements, however, high computation cost presents a significant challenge for such adoptions. Fortunately, commodity graphics processing units (GPUs) that support massive parallel computing power at a very affordable cost have become available over the past few years and there is astounding growth in the use of GPUs for overcoming the computation challenges in these accurate image reconstructions [24]–[28]. In this article, we develop a fast GPU-based algorithm for a high sensitivity small-animal PET as outlined in the following two paragraphs.

Although current dedicated small-animal PET systems have reached good spatial resolution of 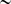
1.2 mm, their sensitivity remains unsatisfactorily low, typically below 5% [29]–[31]. We have recently developed a prototypical small-animal PET (microPET) scanner that can yield a high sensitivity of 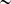
28% when using a 250–750 keV energy window [7]. This scanner employed two detector heads of the HRRT in a stationary, compact geometry to provide both a high intrinsic detection efficiency and a large detection solid-angle. Its compact geometry, however, led to substantial DOI blurring and hence degraded image resolution. The resolution degradations were corrected for in image reconstruction by accurately modeling the scanner's system response matrix (SRM) and incorporating it into an ordered subset expectation maximization (OSEM) algorithm [32] to recover the estimated intrinsic resolution of the HRRT detectors, which is 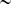
1.2 mm. As we will discuss more clearly below, the developed reconstruction algorithm was computationally very expensive. It needed to handle a linear system matrix that involved more than 200 million measurements and more than 10 million unknown image voxels. In our initial implementation on a CPU system (four Athlon x64 2.0 GHz PCs), the reconstruction generally took days to complete [33]. This long reconstruction time has prevented the routine use of this high-sensitivity scanner despite of our biologist collaborators' high interest for using it.

This paper is therefore concerned with developing a GPU-based algorithm to significantly speed up the reconstructions of our high-sensitivity small-animal PET scanner. Our implementation exploits the symmetry properties in our scanner to take advantage of the parallel computing power of a GPU based on the NVIDIA Compute Unified Device Architecture (CUDA). In particular, we propose a shift-invariant LOR-GPU block mapping scheme and exploit the symmetry properties on GPU warp/thread assignments that result in efficient parallelization with reduced memory access cost. We also take advantage of texture memory for fast random data read, shared memory for parallel reduction summation and data reuse, coalesced global memory access, and atomic operation for avoiding race condition. The developed algorithm is applied to simulated and experimental data and yields results with satisfactory image quality and substantially reduced computational times. It is mentioned that there are also efforts in speeding up reconstruction by approximating the SRM with a simplified model and factorizing the SRM into component blurring processes [34], [35]. Our present implementation does not exploit these approaches but employs a non-factorized, prestored SRM that was accurately calculated by using Monte-Carlo simulations.

In the following sections, we will briefly review the background materials and discuss how to employ GPU computing to accelerate our reconstruction algorithm. A computer-simulation study was carried out to investigate and evaluate the CUDA reconstruction method. The numerical results of which, together with a real-data result, are presented. Finally we conclude the article with a discussion and summary.

## Background Review and System Description

We review the GPU technology as well as the design of our dual-head small-animal PET (DHAPET) scanner as follows.

### GPU architecture

Our reviews first focus on the GPU architecture related to our implementations. In particular, we use NVIDIA Tesla C2070, code named “Fermi”, in conjunction with CUDA, which is an extension of the standard C/C++ programming languages with single instruction multiple threads (SIMT) execution model. Readers who wish to learn more about this subject are referred to many monographs, such as [24], [36], which cover the subject in-depth.

**Figure 1:**
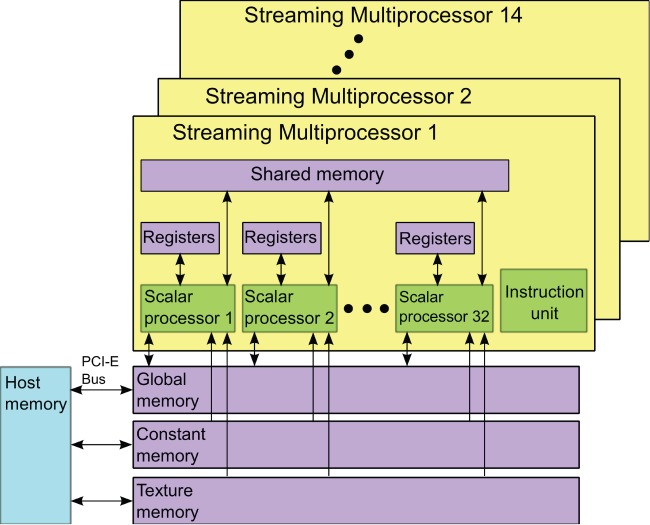
A schematic of the architecture of an NVIDIA Fermi GPU.

A Fermi GPU, illustrated in Figure 1 for its processor and memory schema, contains 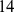 Streaming Multiprocessors (SMs) and Global Memory that can be accessed by all SMs. Globally accessible but read-only Constant Memory and Texture Memory, can be used to accelerate data accesses. The use of Constant and Texture memory can be further accelerated when all threads of a warp (to be explained below) access the same address (i.e., broadcasting) and when memory access patterns exhibit a large degree of spatial locality. Each SM features 32 Scalar Processors (SPs) and a Shared Memory that can be accessed by all 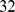 SPs. Each SP also has its own Registers to provide the fastest access to a small amount of data and Instruction Units to increase arithmetic density.

In CUDA programming, the data to be processed on GPU will be transferred from CPU memory to the global memory of GPU first. A CUDA kernel containing a grid of threads is then launched. These threads in the grid are further divided into blocks that are assigned to execute resources of a Fermi GPU. These blocks are implemented independently and each of which is assigned to one SM for execution. Each block is executed as 32-thread warps. At any given time, the SM concurrently executes the 32 threads in only one warp while holding other warps on wait. Those warps that have operands ready for the next instruction are qualified for execution. The switches among warps are fast. When data sharing is necessary, the threads within a block can communicate with one another via the shared memory of the SM to which that the block is assigned.

Another issue that needs be considered in a multi-threaded program is the race condition, which occurs when multiple threads attempt to access the same piece of memory simultaneously and thereby impact the correctness of the result. To avoid race condition, the Fermi GPU provides *atomic operation* to guarantee that at any given time only a single thread has access to a given memory. Invoking atomic operation however can slow down the operations.

Generally, to maximally utilize the parallel computing power of GPU, one should try to maximize independent task/data/thread parallelism, to maximize arithmetic intensity, to perform more parallelizable computations on the GPU rather on the CPU and to avoid data transfer between CPU and GPU. When data accesses are necessary, we should optimize for coalescing in global memory, for spatial locality in texture memory, and for avoiding bank conflicts in shared memory. However, for a specific algorithm implementations of the general rules are not necessarily a trivial task.

### Dual-head small-animal PET scanner

**Figure 2:**
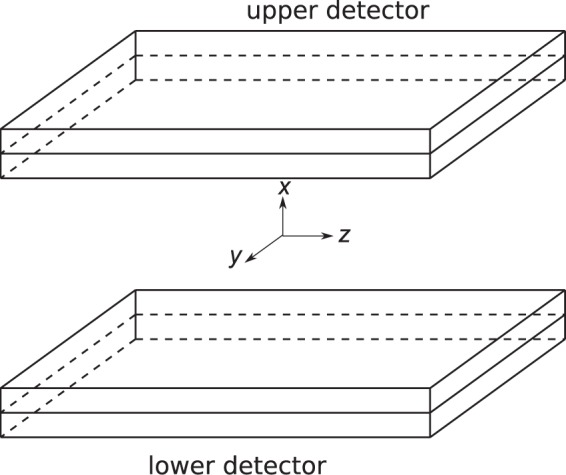
Conceptual illustration of the dual-head PET system with the definition of the coordinate system. The origin is at the center of the active imaging volume, the 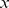-axis is perpendicular to the detectors, and the 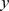
- and 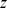-axes are along the length and width of the detectors, respectively.



Our DHAPET scanner, illustrated in Figure 2, consists of two HRRT detectors that have a detection-active area of 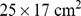. The detectors are placed at only 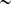
6 cm apart, thereby creating a large detection solid-angle for small animals placed in between [37], [38]. Each HRRT detector is comprised of a 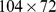 array of 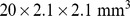 double-layered LSO/LYSO crystals having a crystal thickness and a pitch equal to 20 mm and 2.4 mm, respectively. The scanner remains stationary during imaging. Every pair of two crystals, one from each detector, defines one line-of-response (LOR) and one measurement. Let 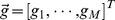 denote the measured data, where 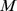 is the total number of LORs of the scanner, and 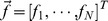 the unknown source image, where 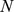 is the total number of image voxels. Let 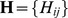 denote the SRM, where 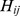 is the probability of an annihilation occurring inside the 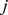
th voxel to be detected at the 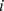
th LOR. The PET imaging model is then given by
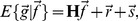
(1)where 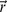 and 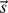 are the mean values of the random and scattered events. In addition, the measurement contains noise and is governed by the Poisson distribution, given by

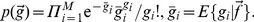
(2)The imaging model given above by Eqs. (1) and (2) can be solved by a number of iterative algorithms. The focus of the present work is to correct for the DOI blurring; therefore, we will ignore random and scattered events and consider solving the imaging model by using the popular OSEM algorithm. By dividing the LORs of the scanner into a number of disjoint subsets, say 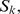 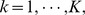 where 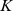 is the number of subsets, and given an initial estimate for 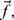 say 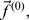 the OSEM algorithm is given by: for 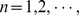


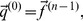
(3)

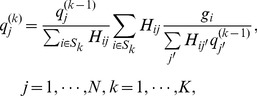
(4)

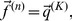
(5)where 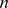 is the iteration number. Unless mentioned otherwise, we will use 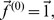 i.e., all voxels of 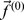 are set to a constant value of 1. By modeling the DOI blurring in the SRM, the reconstruction algorithm can correct for DOI blurring to yield image of good resolution. Readers are referred to [7] for detailed discussion of the design considerations, image reconstruction, and performance evaluations of the DHAPET scanner.

A particular challenge in implementing the above OSEM reconstruction algorithm arises from the extraordinary dimensions of the SRM. The DHAPET scanner contains more than 224 million LORs and more than 14 million voxels when employing a 6 cm detector spacing and approximately 0.5 mm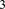 image voxel. As a result, the SRM has more than 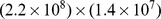 elements and, by brute force, its calculation is extremely challenging, if not impossible. In addition, the forward projector (i.e., the operation 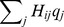) and backward projector (i.e., the operation 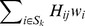 and 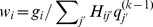) in Eq. (4), and hence the OSEM algorithm, are also computationally very intensive. In [7], we were able to exploit the symmetry properties of the DHAPET to make it feasible to employ Monte-Carlo (MC) calculations to estimate the SRM including the DOI blurring. However, even with the prestored SRM and exploiting the scanner symmetries to speed up the matrix operations that represent the forward and backward projections, the resulting OSEM algorithm still requires more than 20 hours to run one iteration [33]; consequently, it is difficult to employ the DHAPET scanner for routine small-animal imaging studies. The objective of this work is therefore to exploit GPU computing to massively parallelize, and hence drastically accelerate, the reconstruction algorithm.

## Methods for Accelerating the Reconstruction Algorithm by GPU

This section describes our implementation of an OSEM algorithm for the DHAPET scanner on a Fermi GPU. The implementation matches the GPU architecture to the symmetry properties of the scanner and carefully considers the execution order to minimize the slower memory copy operations.

### Symmetry properties

The symmetry properties of the DHAPET scanner, which have been previously described in [39], are summarily reviewed below for completeness.

Refer to Figure 2 for the definition of the coordinate system. Although the HRRT detector contains double-layered crystals, we will consider only single-layered crystals for simplicity. A crystal in an HRRT detector can be identified by 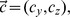 where 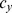 and 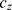 are the row and column numbers of the crystal, respectively. An LOR, or a crystal pair, can then be identified as 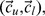 where 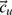 and 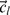 identify a crystal on the upper and lower HRRT detectors, respectively. Similarly, an image voxel can be identified by 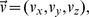 where 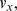 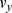 and 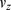 are the slice, row, and column numbers of the voxel, respectively. The detector response function (DRF) 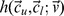 is defined as the probability for an annihilation taking place in voxel 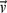 to be detected at the LOR 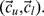 Clearly, 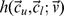 is an element of the SRM 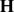 given above in Eqs. (1)-(5). Without loss of generality, we can assume the detector to have a crystal pitch equal to 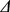. When choosing the voxel size to be 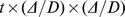, where 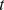 is the voxel thickness in the 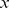 direction and 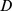 is a positive integer, we have the following symmetry properties:

1. *Shift invariance*: Ignoring boundary condition due to the finite size of the detector, we have
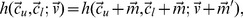
(6)where 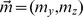 and 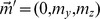 with 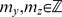.
2. *Reflection symmetry*: Let 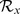, 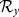, and 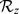 denote the operators that negate the 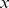, 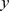, and 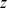 components of its operand, respectively, e.g., 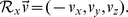 The invariance of the system under reflection with respect to the coordinate axes then implies

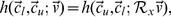
(7)and
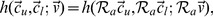
(8)


with 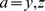.

1. *Axis interchangeability*: Similarly, the system response is invariant when the 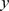
- and 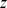-axes are exchanged. Let the operator 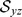 swap the 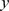
- and 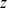
- components of its operand, e.g. 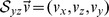. Then,

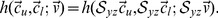
(9)


### Computation of the system matrix

To obtain the best reconstruction results, it is of great interest to eliminate any pre-reconstruction data interpolation or rebinning. We employed the GATE package [40] to accurately model the detection characteristics of the HRRT detector heads for the dual-head configuration [41]. The GATE package is a public-domain MC simulation package that can accurately model the physical processes and geometric effects [42], [43].

In this work, we consider the following settings. Two detector heads with the crystal pitch size of 2.4 mm were positioned at 6 cm apart. We chose 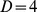 and 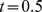 mm to obtain a voxel size of 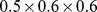 mm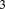. We also chose to have 119 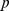-planes and positioned the central plane at exactly the midway between the detectors, thereby making 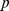-planes symmetric with respect to the reflection about the 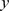-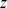 plane (see Symmetry properties section). GATE package generated the point spread function (PSF) for every three seed voxels at each 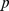-plane. In our MC simulation, these seed voxels were filled with a uniform activity of a pair of back-to-back 511 keV 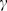-photons, and a 350–750 keV energy window and a 6 ns coincidence time were assumed. By using the high symmetry properties of the dual-head PET, we can specify “one” LOR or the DRF to compute its response to the uniform activity distribution in the whole imaging space. As a result, to construct the SRM, we only need to compute the detector response functions for 60 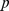-planes, whose index values are within the range of [0 59] and 3 voxels in each 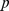-plane [7]. The detector head has 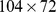 pixels and was extended to 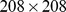 to enable the use of symmetries to be applicable to all elements in the DHAPET system matrix. We exploited the symmetry property and computed the system response matrices, which were stored as sparse matrices to facilitate access in reconstruction. Note that the subject scattering, positron range and photon linearity are not included. Also, the random events were excluded and the scanner dead-time was ignored.

### GPU parallelizations

The OSEM algorithm for planar PET can be accelerated by taking advantage of the abundant GPU threads. The computation of forward or backward projection involves multiplication of SRM with a large number of voxels or LORs; therefore, it is natural to assign one thread for each voxel or each LOR. However, such a straightforward GPU parallelization is not optimal as it will yield extremely long reconstruction times. To demonstrate the impact of parallelization schemes, we developed three GPU algorithms using different acceleration strategies. The ideas of the three strategies are presented below.

• **Algorithm 1. Forward projection using one thread block for one LOR.** In this straightforward GPU parallelization, we do not exploit the symmetry properties of DHAPET nor do we use particular memory usage schemes in GPU. The step-by-step procedure is shown below.
  - 1: **allocate** a 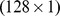 thread block to handle 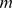
th LOR
  - 2: thread[i] reads the corresponding f[i][m] in the 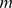
th LOR from the global memory simultaneously
  - 3: thread[i] computes 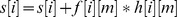 simultaneously and stores it in its register
  - 4: **for** 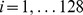 **do**
  - 5: proj = proj+s[i] thread[0] sums over 128 threads to obtain forward projection value
  - 6: **end for**
• **Algorithm 2. Forward projection using one thread block for multiple reflection and axis symmetric LORs.** As shown in the previous “Symmetry properties” section, the geometric symmetries in the dual-head PET setting include shift invariance, reflection symmetry, and axis interchangeability. This algorithm integrates these symmetry properties with GPU architecture to expedite the image reconstruction. The step-by-step procedure is shown below.
  - 1: **allocate** 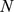 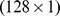 thread blocks to handle the 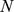 shift-invariant LORs
  - 2: **for** 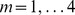 **do**
  - 3: thread[i] reads the corresponding f[i][m] in the 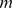
th symmetric LOR from the texture memory simultaneously
  - 4: thread[i] computes 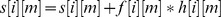 simultaneously
  - 5: thread[i] stores s[i][m] in the global memory simultaneously
  - 6: **end for**
• **Algorithm 3. Forward projection using one thread block for multiple reflection and axis symmetric LORs, as well as shared memory for fast data read and write.** Because the memory hierarchy in GPU is notably different from that in CPU, this algorithm carefully considers GPU memory structure and suitably uses faster accessing memory like texture memory, shared memory, and parallel reduction scheme. The step-by-step procedure is shown below.
  - 1: **allocate** 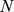 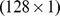 thread blocks to handle the 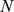 shift-invariant LORs
  - 2: **allocate** 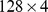 block shared memory for storing partial sums
  - 3: **allocate** 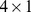 block shared memory for storing histograms of 4 symmetric LORs
  - 4: **for** 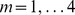 **do**
  - 5: thread[i] reads the corresponding f[i][m] in the 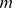
th symmetric LOR from the texture memory simultaneously
  - 6: thread[i] computes 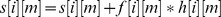 simultaneously
  - 7: thread[i] stores s[i][m] in the blocked shared memory simultaneously
  - 8: **end for**

In the next two sub-sections, we discuss how the symmetry properties can be integrated with GPU and how the GPU memory hierarchy can be efficiently used.

### Integration of symmetry properties with GPU architecture

We have reviewed GPU architectures, OSEM algorithm for PET image reconstruction, and symmetry properties in our system. In this section, we will describe our strategies to accelerate image reconstruction: (i) efficient arrangements of blocks, warps, and threads toward algorithmic structure of the OSEM algorithm and the symmetry properties of the DHAPET scanner, (ii) efficient GPU memory usages, and (iii) other efficient implementation considerations. Note that as mentioned above, the SRM is pre-calculated. Even so, the OSEM algorithm is still computationally very expensive due to the large numbers of LORs and image voxels involved. The planar geometry of two 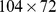 detector arrays yields LORs with a total of 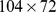 different directions, which are randomly divided into 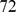 subsets. Each subset, containing 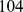 LOR orientations, is accompanied by a seed-DRF pool file that specifies the memory addresses of sensitivity functions associated with voxels intersected by these LORs.

First, we highlight how the parallel computations of shift-invariant LORs can be performed efficiently via the abundant CUDA blocks, warps, and threads and how the cost to access SRM can be reduced.

• *A shift-invariant LOR-block mapping yields 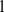 to 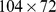 independent tasks being executed simultaneously*.

**Figure 3:**
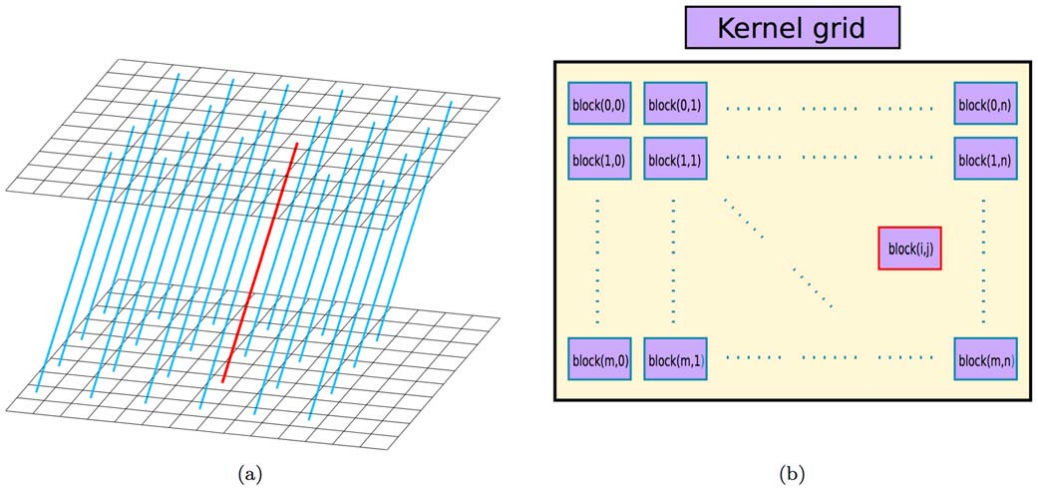
A shift-invariant LOR-block mapping schema. Each shift-invariant LOR shown in (a) is associated with a GPU block shown in (b). Note that only partial LORs are shown in subfigure (a) to avoid cluttering the plot.

In both forward and backward projections, multiple CUDA blocks were assigned to compute the same number of shift-invariant LORs for a given orientation, as their computations are independent of one another. For example, as conceptually shown in Figure 3, these LORs are shifted from the perpendicular direction by 2 elements in both 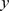
- and 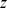
- axes in the upper detector so that there are 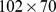 LORs of such an orientation and thus their forward projections will be determined by the same number of CUDA blocks.

• *Exploiting the reflection symmetry and axis interchangeability on warp/thread assignments leads to a 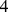 to 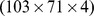-fold saving for one LOR orientation in the SRM accessing times.*

**Figure 4:**
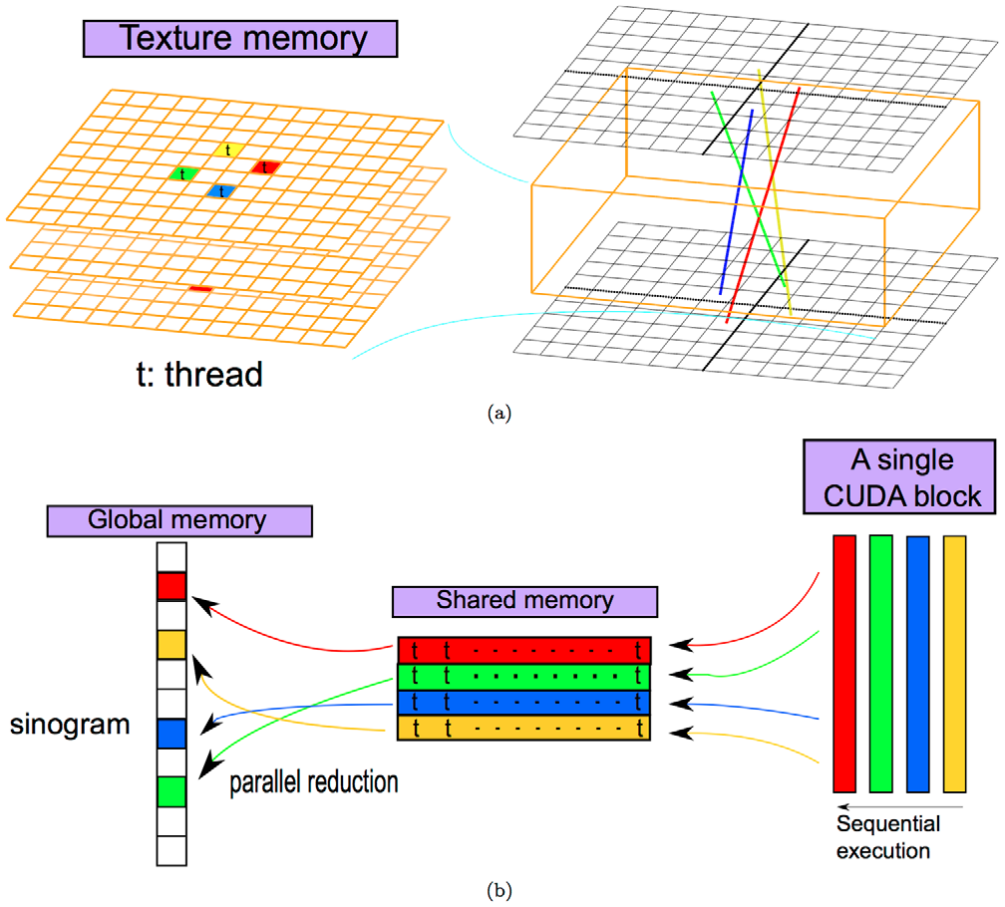
The proposed GPU strategies. (a) The active imaging volume stored in the global memory is bound with the texture memory. The LOR of a particular direction (red) and its symmetric equivalents corresponding to reflection symmetries in 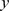-axis (yellow) and 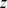-axis (blue) and interchanged 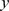
- and 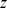-coordinates (green) are shown in the right panel. (b) Each CUDA block contains 128 threads (four warps). These threads multiply the sensitivity functions with the corresponding voxels and store the values in their shared memory for each LOR sequentially. The accumulative summations in the forward projection is performed on the shared memory with parallel reduction summations.

Four-fold and 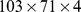-fold speedups arise from the fact there are 4 most oblique LORs and 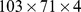 LORs that are shifted from perpendicular direction by 1 element in both 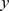
- and 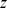
- axes, respectively. Within each block, the reflection symmetries in 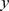
- and 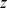
- axes and axis interchangeability between 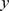
- and 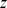-coordinates were further exploited to yield additional 4 symmetric LORs, which were computed using 128 threads. Figure 4 illustrates the LOR of a particular direction in red and its symmetric equivalents corresponding to reflection symmetries in 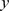
- and 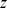-axes and interchanged 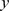
- and 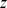-coordinates are depicted in yellow, blue and green, respectively. The application of these symmetry and the shift-invariant properties accounts for a 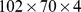-fold saving in the SRM accessing times for the orientation shown in Figure 4 by using the sensitivity functions common to these LORs of the given orientation.

The exploitation of shift-invariant and reflection symmetry and axis interchangeability for all LORs of orientations amount to 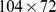-fold saving in the SRM accessing times.

### Efficient memory hierarchy for forward/backward projections

Second, as the bottleneck of the OSEM in the planar PET geometry is the memory bandwidth rather than the floating point arithmetic operations, we emphasize how we can utilize the various GPU memory efficiently in the OSEM algorithm.

**Figure 5:**
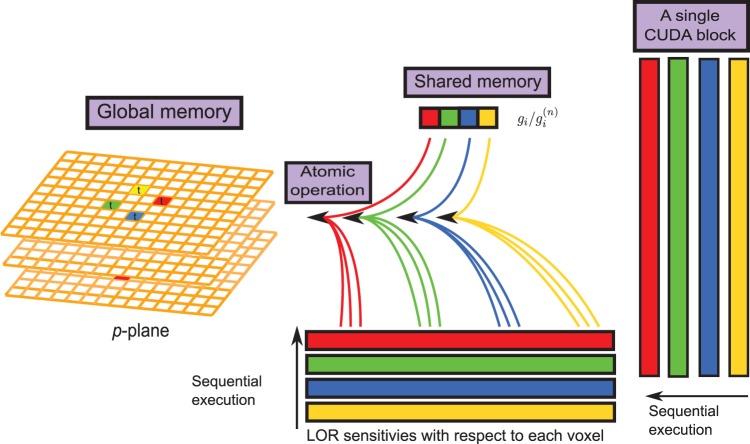
The schema of the backward projection. A block of 128 threads read 
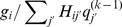
 from their shared memory, multiply it with sensitivity functions and then backproject to the corresponding voxels intersected by each LOR sequentially.

• *Texture memory binding for fast random data read*. In forward projection, the volume estimate 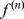, which is stored in the global memory was bound with texture memory to facilitate random data access for forward projections, as shown in the left panel of Figure 4. Taking advantage of the high efficiency in memory access, the data could be read from texture memory swiftly.
• *Shared memory coalescing for parallel reduction summation*. The data related to 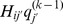 in forward projection is stored in the shared memory, as shown in Figure 4(b). Subsequently, memory coalescing can be achieved by the parallel reduction summation [44], which was applied to accelerate the accumulative summations in the forward projection. Our implementation was modified from the CUBLAS isamin function.
• *Shared memory for data reuse*. In the forward and backward projections, we employed the first threads of 4 warps (threads 0, 32, 64, 96) within each one-dimensional CUDA block (128 threads) to allocate the histogram data of 4 symmetric LORs to the shared memory. In lieu of assigning a single thread to execute the instruction sequentially or invoking 4 threads in the same warp to access 4 elements that reside on non-contiguous addresses, executing other warps can hide latencies and keep the hardware busy. As indicated by Figure 5, these 4 threads belonging to 4 different warps also allocate the ratios of measured LORs to the projected ones (i.e., 
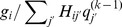
) at the 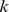-th subset to their shared memory for backward projections. Subsequently, these ratios are retrieved and multiplied with the sensitivity functions before backprojecting to the corresponding voxels.
• *Global memory without race condition*. When writing the forward results to the global memory, the atomic operator atomicAdd was applied to avoid race condition. Similarly, before the operand of each thread was backprojected to the global memory, atomicAdd operator was also applied to ensure that no race condition occurs.

Third, some other acceleration techniques are remarked.

• Before the forward or backward projections along each LOR was carried out, all threads in the block will determine whether the measured histogram data stored in shared memory contain nonzero values. If the data value is zero (i.e., no signal), we assign zero to the corresponding value without further computation.
• We can avoid memory access by recomputing indices on the fly, as memory access can take more time than recomputing.

In summary, our GPU acceleration schemes utilized the LOR-based DRF and exploited the abundant threads, spatial locality and other features of the GPU architecture to significantly accelerate both the forward and backward projections. As shown in the next section, the GPU codes achieve significant speedups and thereby largely improve the applicability.

## Reconstruction Results

### Image reconstruction

A simulated and real datasets were used for evaluation. For the simulated dataset, we implemented a numerical version of the micro Deluxe Derenzo phantom. The phantom was placed at the center of the scanner with the rods positioned along the 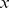-direction (i.e., vertical to the detectors). We first applied the pre-calculated SRM for the scanner to this numerical phantom to generate noise-free measurements. Then, Poisson noise is introduced to obtain a simulated dataset containing a total of 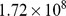 true events.

**Figure 6:**
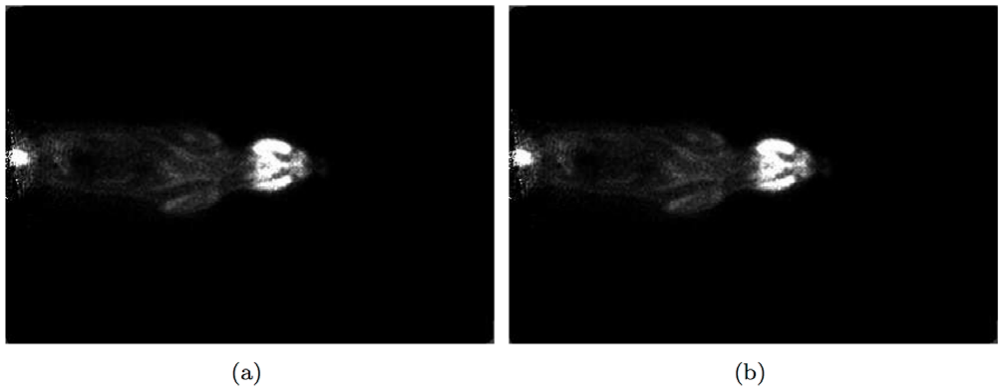
A sample coronal slice of the reconstructed image of a real dataset acquired for a healthy adult rat injected with 18-FDG by the DHAPET scanner, by using either (**a**) **the CPU- or** (**b**) **the GPU-based OSEM algorithm.** The head of the rat was placed at approximately the center of the scanner. The images generated by the two algorithms show negligible differences.

The real dataset was obtained by imaging a healthy adult rat (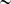
270 g) by the DHAPET scanner. The rat was placed with its head approximately at the center of the scanner. A bolus injection of 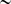
500 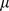
Ci of 18-FDG via the tail vein was administrated and the rat was scanned immediately after the injection for 30 minutes. Random correction was performed by subtracting the delayed coincidence and removing any resulting negative measurements. The resulting dataset contained about 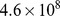 counts. It was then normalized for the variation in sensitivity across LORs using measured normalization factors. No correction for subject attenuation or scatter was performed. Figure 6 shows a sample coronal slice of the reconstructed image of the rat dataset by the DHAPET scanner using (a) CPU- and (b) GPU-based OSEM algorithms, respectively. Both datasets contained coincidence events that only involved the front-layer crystals.

Image reconstruction by use of CPU code was performed on a workstation equipped with an Intel Intel Xeon-E5620 quad-core CPU at 2.40 GHz and 96 GB main memory. An NVIDIA Tesla C2070 GPU with 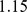 GHz cores and 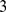
GB on-board memory is installed in the workstation. The CPU code was compiled by using the Intel C compiler icc. The GPU code was compiled by using Intel icc version and NVIDIA CUDA compiler [45]. In all compilations, we use optimization level -O3. The same extended matrix and symmetry properties are used for both CPU- and GPU-based approaches.

**Figure 7:**
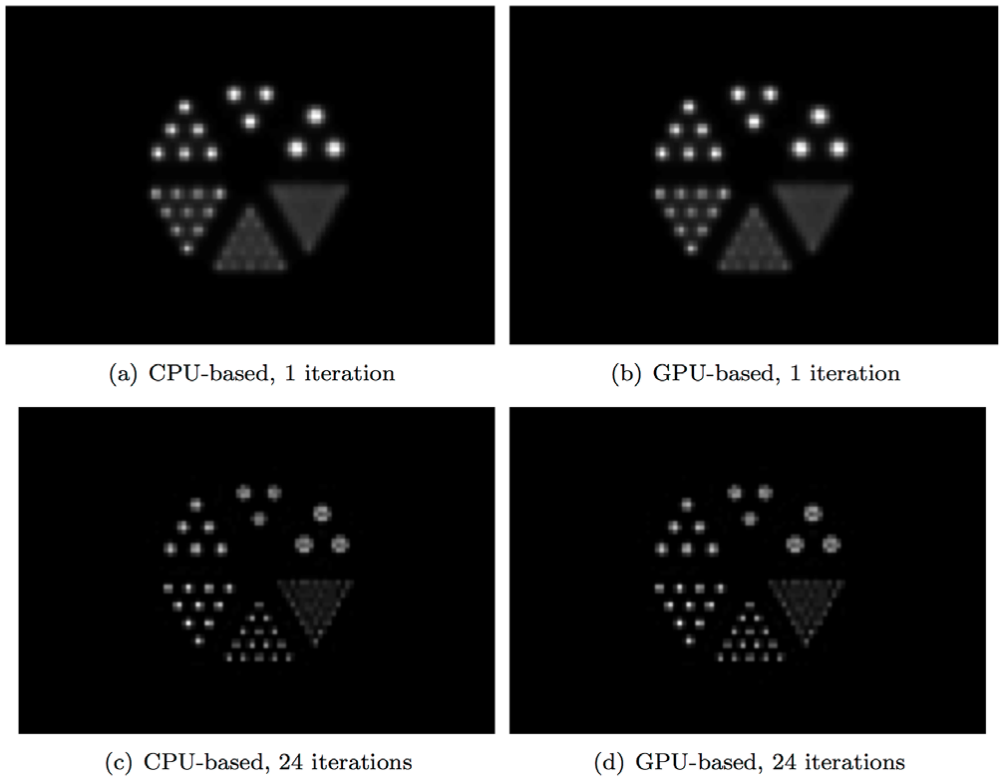
Reconstructed images obtained from simulated data for the numerical micro Deluxe Derenzo phantom by use of CPU- or GPU-based OSEM algorithm after 1 or 24 iterations. The phantom contains 6 groups of rods of different diameters, including 2.4 mm, 2.0 mm, 1.7 mm, 1.35 mm, 1.0 mm, and 0.75 mm. The spacing between the rods in the same group is twice their diameter. The phantom is placed at the center of the scanner with the length of the rods oriented along the 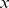-axis. As shown, the images generated by the two algorithms have negligible differences.

**Figure 8:**
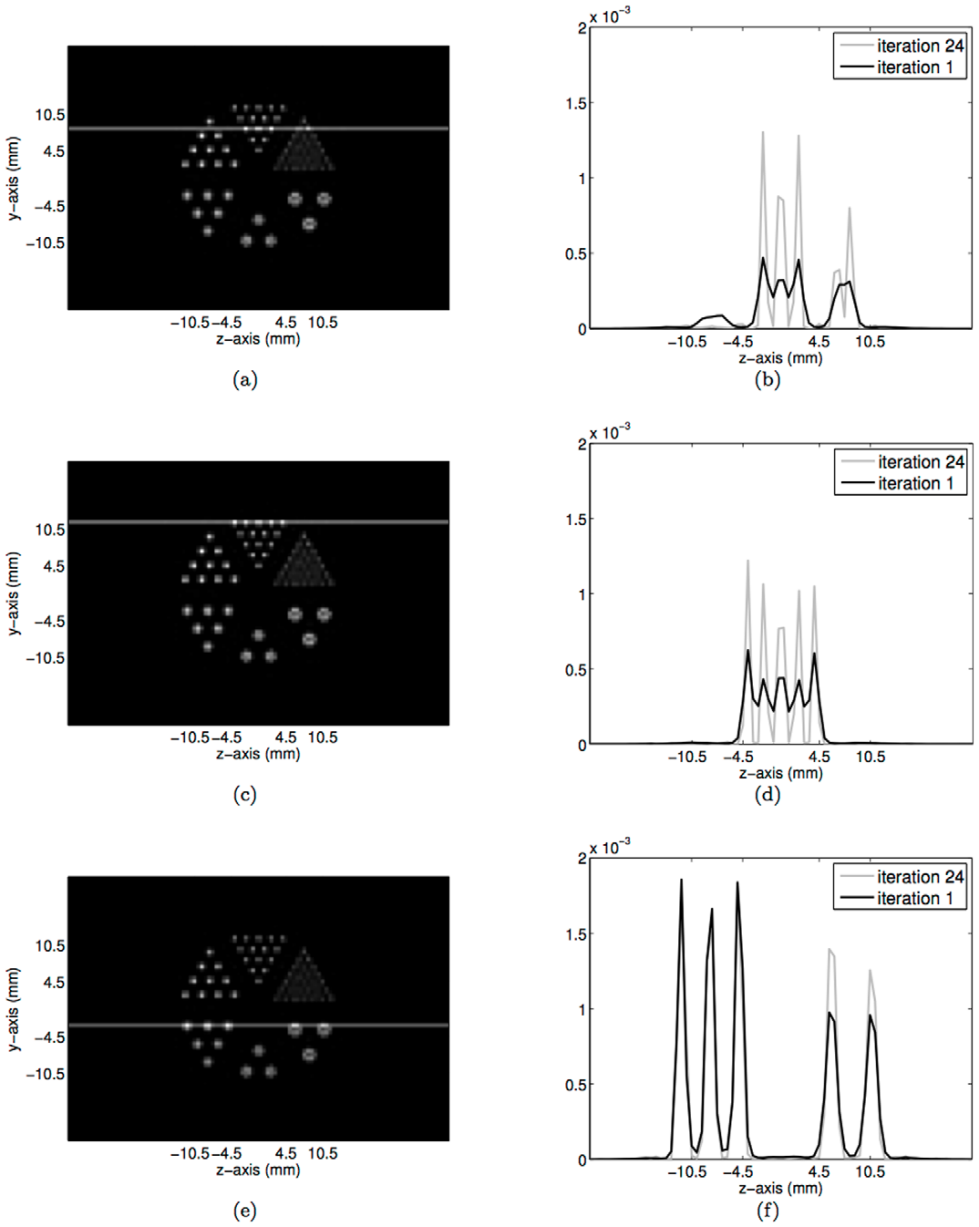
Intensity profiles of the reconstructed images of the numerical micro Deluxe Derenzo phantom at three selected horizontal positions as identified on the image obtained with 24 iterations. Evidently, small rods of the phantom are much better resolved and show much higher contrast, with 24 iterations.

**Figure 9:**
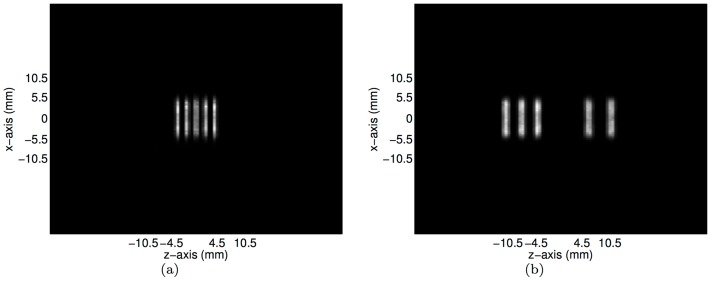
The transverse slices of the reconstructed images of the numerical micro Deluxe Derenzo phantom at (**a**) 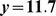 **mm and** (**b**) 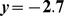 **mm as the horizontal lines identified in** **Figures** **8**(**c**) **and 8**(**e**)**, respectively.**

Both the CPU- and GPU-based OSEM codes have successfully reconstructed the testing images. Visually, and confirmed numerically, differences between the images produced by the CPU- and GPU-based OSEM algorithms are negligible, validating our GPU implementation. Figure 7 shows the reconstructed images obtained from simulated data for the numerical micro Deluxe Derenzo phantom by use of CPU- or GPU-based OSEM algorithm after 1 or 24 iterations. As shown, the images generated by the two algorithms have negligible differences. Figure 8 compares the intensity profiles of the micro Deluxe Derenzo phantom after 1 and 24 iterations. As shown, the small rods of the phantom are much better resolved with 24 iterations. However, with the CPU-based algorithm one iteration requires about 20 hours of computation; in routine use, one therefore would be constrained to perform no more than a few iterations and to accept sub-optimal imaging results. To show homogeneity and resolution of the reconstructed images along 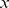-axis, the transverse slices at 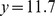 mm and 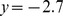 mm are plotted in Figures 9(a) and 9(b), which correspond to the horizontal lines identified in Figures 8(c) and 8(e), respectively.

### Performance evaluations

The choice of algorithms has significant impact on the resulting computational times. To demonstrate such effects, we have performed numerical experiments by using all three algorithms introduced in the sub-section “GPU parallelizations”. Table 1 listed the estimated computation times for forward projection corresponding to Algorithms 1, 2 and 3. Algorithm 1 took around 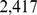 seconds to implement one iteration by computing 1 LOR with one 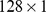 thread block. Although the use of symmetry properties in Algorithm 2 can significantly reduce the reconstruction times to 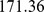 seconds, it is still much longer than the 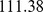 seconds achieved by Algorithm 3. Further, we implemented Algorithm 3 by using both 1 and 128 threads to investigate the impact of thread number. The GPU forward projection time per iteration was found to increase from 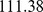 to 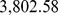 seconds when the thread number reduced from 128 to 1.

**Table 1:** Computational time for the forward projections in the OSEM algorithm (in seconds) using different GPU algorithms.

	Algorithm 1	Algorithm 2	Algorithm 3
Acceleration strategies	No symmetry properties, no shared memory	Use symmetry properties	Use symmetry properties and memory allocation
Time (s)	∼2,417	171.36	111.38

Tables 2 and 3 summarize the timing results obtained for the CPU- and GPU-based algorithms. When applied to the simulated dataset, the CPU and GPU codes take, respectively, 14,312 seconds and 111.38 seconds to compute the forward projection per iteration, or 198.78 seconds and 1.55 seconds per subset on average. This represents a factor of 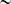
128 in speedup. In comparison, the CPU and GPU codes take respectively 23,873 seconds and 678.27 seconds to compute one iteration of the backprojection, or 331.57 seconds and 9.42 seconds per subset on average. In this case, about a factor of 35 in speedup is realized. Note that for both CPU and GPU codes the backprojection is computationally more expensive than the forward projection. This difference is related to the different memory-access patterns of our implementations of the forward and backward projections. When applied to the real rat data (see Table 2), both the forward and backward projections require slightly longer computation time. The speedup factor for forward projection is also slightly reduced to 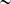
120, but the speedup for the backward projection remains essentially identical at 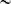
36.

**Table 2:** Computation time of the OSEM algorithm (in seconds) and speedup by the GPU implementation based on the simulated data of the micro Deluxe Derenzo phantom.

	Forward projection per subset	Forward projection per iteration	Backprojection per subset	Backprojection per iteration
CPU	198.78 (1X)	14,312.00 (1X)	331.57 (1X)	23,873.00 (1X)
GPU	1.55 (128.24X)	111.38 (128.50X)	9.42 (35.20X)	678.27 (35.20X)

**Table 3:** Computation time of the OSEM algorithm (in seconds) and speedup by the GPU implementation based on the rat dataset.

	Forward projection per subset	Forward projection per iteration	Backprojection per subset	Backprojection per iteration
CPU	246.68 (1X)	17,761.00 (1X)	376.96 (1X)	27,141 (1X)
GPU	2.06 (119.82X)	148.23 (119.82X)	10.55 (35.72X)	759.80 (35.72X)

We have also evaluated our GPU codes by using the CUDA Visual Profiler version 4.0.17 and the results indicate that the implementation is efficient. First, we look at the occupancy metric that is computed by taking the ratio of the active thread numbers to the maximum thread numbers per processor. While the theoretical occupancy equals 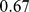, the forward and backward projections can achieve occupancy that are as high as 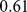 and 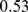, respectively. The backward projection has lower occupancy as it takes more time on writing data to global memory. Second, we focus on the GPU utilization percentages. The kernel time occupies 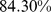 out of the total GPU time, while memory copy time occupies only 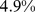 of the total GPU time. This observation suggests that our codes can successfully avoid relatively expensive (compared with data accesses on GPU) data transfer between the CPU and GPU and devote most of it resources in the computation part.

## Discussion and Summary

We applied similar acceleration strategies to both forward and backward projections; however, the computation of the latter is considerably longer than the former due to the fact that the data structure of the SRM permits only the LOR-based projections. The excessive computational time results from the random writing to the memory in the backprojection for both CPU- and GPU-based codes. The CPU computational times was, in fact, already shortened because there exist a great deal of LORs in the DHAPET system that are shift-invariant or symmetric in some direction. This unique feature in the panel PET system allows for the direct mapping of parallel LORs to the CUDA block and thread units in GPU to facilitate parallel computing. In addition, the multi-threaded program can be further parallelized by employing multiple GPUs. We have demonstrated that the parallel computing units and GPU memory can be integrated with the data structure of the DHAPET system to deliver the image reconstruction in a timely manner. Recently, the hardware advancement driven by the demands for a better imaging performance leads to dedicated animal PET systems with good spatial resolution and finds new PET applications in the fields of food science and plant imaging. The quantitative information of physiological effects and translocation of bioactive ingredients provides new insights on food chemistry and mechanisms of reactions like photosynthesis and photo-assimilation [46]–[48]. In addition to providing high detection sensitivity and cost effectiveness, planar PET imaging is particularly suitable for imaging thin and small objects like plant leaves. The growing interest in these extended applications also inspired development of PET detectors designed specifically for plants [9], [10]. Our algorithm can facilitate a wider range of applications for dual-head PET systems, and can be generalized to accommodate more scanning geometries.

We have applied GPU architecture in our GPU-based algorithm to remarkably accelerate the OSEM reconstruction in our dual-head PET system. Numerical studies of the developed algorithm were conducted to demonstrate the achievable speedup employing the currently available GPU hardware. The multi-threaded units, various memory types and other unique features of GPU can be exploited to expedite image reconstruction drastically. With the substantial speedup by the GPU code, we would be able to routinely employ 10 or more iterations in reconstruction to achieve better image resolution and contrast.
